# The role of tissue-resident memory T cells as mediators for response and toxicity in immunotherapy-treated melanoma—two sides of the same coin?

**DOI:** 10.3389/fimmu.2024.1385781

**Published:** 2024-03-18

**Authors:** Robin Reschke, Benjamin Deitert, Alex H. Enk, Jessica C. Hassel

**Affiliations:** ^1^ Department of Dermatology, National Center for Tumor Diseases Heidelberg (NCT), Heidelberg, Germany; ^2^ Institute for Tumor Biology, University Medical Center Hamburg-Eppendorf, Hamburg, Germany

**Keywords:** TRM cells, tissue resident memory T cells (TRM), irAE, immune related adverse effects (irAEs), immunotherapy, biomarker, immune checkpoint inhibitor (ICI), melanoma

## Abstract

Tissue-resident memory T cells (T_RM_ cells) have become an interesting subject of study for antitumor immunity in melanoma and other solid tumors. In the initial phases of antitumor immunity, they maintain an immune equilibrium and protect against challenges with tumor cells and the formation of primary melanomas. In metastatic settings, they are a prime target cell population for immune checkpoint inhibition (ICI) because they highly express inhibitory checkpoint molecules such as PD-1, CTLA-4, or LAG-3. Once melanoma patients are treated with ICI, T_RM_ cells residing in the tumor are reactivated and expand. Tumor killing is achieved by secreting effector molecules such as IFN-γ. However, off-target effects are also observed. Immune-related adverse events, such as those affecting barrier organs like the skin, can be mediated by ICI-induced T_RM_ cells. Therefore, a detailed understanding of this memory T-cell type is obligatory to better guide and improve immunotherapy regimens.

## Introduction

Immunotherapy-based therapies have ushered in an era of unprecedented improvement in the prognosis of malignancies in metastatic states of the disease (1). Although immune checkpoint blockade (ICI) provides a long-lasting response in approximately one-third of melanoma patients, a high number of non-responders and immune-related adverse events (irAEs) need to be considered as well (1). Therefore, a high medical need exists to identify treatment responders and patients with a predisposition for irAEs early on. Recent studies have identified tissue-resident memory T cells (T_RM_ cells), correlating with clinical endpoints such as prolonged survival in different ICI-treated cancer entities (2–6). In particular, CD103^+^CD39^+^ T_RM_ cells were associated with better outcomes across different tumor entities (5). Within this review, we will highlight the functional aspects of T_RM_ cells that underpin their prognostic role in melanoma antitumor immunity and ICI efficacy. However, T_RM_ cells were also shown to be involved in irAEs of barrier organs such as the skin or the gut (7–10). We will elaborate on these off-target effects also.

## Function and genesis of tissue-resident T cells in the skin

Tissue residency and memory functions enable T_RM_ cells to rapidly defend against encountering viral infections and other pathogens these cells are previously primed against (11). In the course of a lifetime, inflammation or exposition to viral infections and microbiota contributes to the genesis of T_RM_ cells and shapes an individual and diverse immunological landscape. Interestingly, T_RM_ cells differ in their mode of action from primary T-cell responses. In their inactivated cell state, T_RM_ cells were reported to pass throughout the tissue and express dendritic-cell-like pseudopods to recognize antigens (11). After pathogen sensing, T_RM_ cells rapidly expand and proliferate, secreting IFN-γ, which evokes local inflammation and CXCL9 and CXCL10 secretion (12). CXCR3 ligands are known to attract more lymphocytes to the tissue which then can also correlate with antitumor immunity and ICI efficacy (13, 14). In mice lacking circulating T cells, T_RM_ cells eradicate pathogens by upregulation of cytotoxic cellular function in an IFN-γ–dependent fashion (15). The functional features of T_RM_ cells are predetermined by the expression profile of the cell: acquiring increased expression of CD69, Hobit, Blimp1, or Runx3 and downregulating CD62L, S1PR1, Tcf1, T-bet, or Eomes (16–19). The cytokine IL-15 induces CD69 expression (8, 20). In the skin, T_RM_ genesis is triggered by inflammation and antigen presentation. In line with these findings, human keratinocytes cocultured with T cells induce CD103 expression by TGF-β, enabling T_RM_ cells to consolidate (21). T_RM_ cells showed more potent effector functions than recirculating T cells (21). Interestingly, T_RM_ and T_CM_ were shown to share the common naive T-cell progenitor/precursor (22). Progenitors of memory cells from the bloodstream extravasate and express CD103, induced by TGF-β (21). Entry into the epidermis is considered an essential step in the maturation of T_RM_ cells identified by upregulated CD69 and the prosurvival marker Bcl-2 (23). In contrast, a comparable set of cells in the dermis was found to be CD103^−^, emphasizing the influence of environmental factors in the epidermis (23).

Induced by viral infection of the skin (modulated by HSV infection of the skin in mouse models), keratinocytes produce CXCL9 and CXCL10, attracting KLGR^+^ T_RM_ progenitors more effectively due to their higher expression of CXCR3 than KLGR^−^ effector cells (23). These cells, after entrance into the skin, are exposed to IL-15 and TGF-β, both known to influence memory T cells toward tissue residency. Interestingly, the longevity of memory T cells seems to be maintained by the KLGR transcription factor, regulating these effects in T_CM_ cells as well as in T_RM_ cells. The plasticity of T_RM_ cells allows them to re-access circulation and differentiate into T_CM_ which patrol lymph nodes (24). This interconversion of memory T-cell phenotype from resident to circulating can be referred to as “outside-in immunity” (24).

Single-cell RNA sequencing and knockout experiments revealed the essential role of transcriptional “master” regulators Runx3, Hobit, and Blimp1 (23). These regulators mediate particular T_RM_ features like TGF-β sensitivity mediated by Smad3, which is positively regulated by Fosl2 or Blimp1 and interacts with Hobit to suppress lymph egress by CCR7 and S1P signaling suppression (23). Circulating memory T cells and tissue-adherent memory T cells differ in their expression profile of these transcription factors. Hobit, for example, was identified in precursors for resident T cells but not in the circulating fraction of T-cell precursors (25). For T_RM_ survival, however, transcription factors like STAT5 and signaling of phosphatidylinositol 3-kinase (PI3K)/Akt and Wnt are mandatory (26). Interestingly, maintenance of T_RM_ cells is achieved by the regulation of metabolic features like fatty acid uptake and beta-oxidation (27). Furthermore, hypoxia was shown to promote and maintain T_RM_ fate (28).

## Immune checkpoint inhibition and tissue-resident memory T cells

Mechanistically, ICI targets a subclass of lymphocytes that express regulatory molecules, called checkpoints. Due to (neo-)antigen overstimulation in the tumor microenvironment (TME), tumor-infiltrating lymphocytes (TILs) acquire a “self-protection” state, referred to as T-cell exhaustion (29). Due to their upregulated checkpoint proteins and their priming against tumor neoantigens, the subpopulation of exhausted T cells (T_EX_) is the target of ICI therapy and increases over the course of therapy, measured by the increase of proliferative markers Ki-67 and PD-1 expressing T cells from 50% prior to therapy up to 75% in the patients’ blood (29).

Elaboration of blood and tissue composition of responders and non-responders could reveal that the antitumor effects of ICI are facilitated by the accumulation of diverse clusters of lymphocytic cells representing adaptive immunity, widely captured as TILs. Distinct blood and tissue characterization further unraveled the subclusters of lymphocytes mediating the response to ICI, identifying memory-like T cells as the most abundant lymphocyte cluster (30). In high-risk patients with melanoma brain metastases, a sequencing scheme of radiotherapy first followed by immunotherapy led to higher frequencies of memory T cells (T_MEM_ cells) in the blood and improved response (31). T_MEM_ cells possess the ability of longevity after (neo-)antigen contact and form thereby a persisting pool of effector T cells facilitating an early adaptive response to previously encountered pathogens. A subset of T_MEM_ cells is tumor-specific and resides within tumor-draining lymph nodes (32). T_MEM_ cells maintain plasticity and can shift from being circulatory T_MEM_ cells to become a T_RM_-like phenotype in melanoma tumors and persist after tumor eradication (33). Batf3-dependent DCs are essential for both memory cell compartments. They are required for the generation of skin T_RM_ cells and they can reactivate circulating CD8^+^ T_MEM_ cells, inducing antitumor immunity (33, 34). Intratumoral T_RM_ cells were expressing PD-1 and expanded after anti-PD-1 therapy, arguing for a key role in ICI efficacy (33). T_RM_ cells are expressing integrins (CD49a and CD103) and losing proteins required for lymph egress (S1PR1 suppressed by CD69). T_RM_ cells represent a subset of TILs with the highest expression of various checkpoint molecules, making them the ideal targets for ICI (35). These cells are under extensive research, considered as secondary acquired defense against pathogens in barrier tissue. Due to their abundance and heterogeneity, memory T cells are thought to substantially contribute to antitumor immunity. Thus, T_RM_ cells are eligible to counteract tumorigenesis and intervene in the different milestones in the metastatic cascade. A plethora of work showed the outgrowth of this fraction of T_RM_-like T_EX_ cells correlating with clinical endpoints like prolonged survival in different ICI-treated cancer entities (2–6). Hence, these cells might hold the potential to eradicate cancerous cells across tumor entities. In particular, skin cancers such as melanoma seem predestined for the already present T_RM_ cells patrolling the tissue and contributing to tumor immunity.

## Tissue-resident memory T cells in melanoma

T_RM_ cells were found to be most abundant in non-lymphatic tissue, rendering them as secondary effectors for bacterial or viral infection (12). For antitumor defense, their characteristics as the predominant T-cell population and their heterogeneity in the T-cell receptor (TCR) repertoire potentially enable effective antitumor responses (35). Residency, mediated by their constitutive expression profile of CD69 and CD103, allows early and immediate recognition and effector function in peripheral tissue, making them presumably important regulators of tumorigenesis. In mice, T_RM_ cells mediated protection against melanoma development (36). CD8^+^ epidermal CD69^+^CD103^+^ T_RM_ cells correlated with spontaneous disease control after inoculation with melanoma cells (36). Mice without T_RM_ cell formation were more susceptible to tumor development (36). In a mouse model analysis, melanoma-specific T_RM_ cells could also be identified in skin-draining lymph nodes (37). These T_RM_ cells protected against melanoma tumor seeding in lymph nodes. T_RM_ cell signatures were also found in sentinel lymph node metastases from patients and correlated positively with survival (37). In a small cohort comparison of four non-responders to four responders in human melanoma, the loss of CD63 and E-cadherin, the target of CD103, was associated with non-response in melanoma (38). E-cadherin resulted in a more mesenchymal phenotype of tumors which were non-responding tumors. In a B16F10 melanoma mouse model, deeper investigations unraveled the association of the loss of E-cadherin and reduced T_RM_ activation in melanoma (39). T_RM_ cells are capable of adhering to epithelial cells and melanoma cells by their CD103 (alphaE) expression (40). CD103 binds E-cadherin by forming a heterodimer with integrin beta7. This homing of T cells provokes a selective pressure for tumor cells to lose E-cadherin to resist reinforced T_RM_ cell immunity (39). Shields et al. showed by implanting E-cadherin overexpressing B16F10 melanoma cells in wild-type, RAG^−^/^−^, and CD103^−^/^−^ mice that E-cadherin expression, which initially led to reduced outgrowth in wild-type mice compared with CDH1 knockout B16F10s, requires functional CD103^+^ T_RM_ cells for successful tumor eradication (39). In a mouse model of adoptive T-cell therapy for melanoma, knockout variants of *Runx3* (identified as one of the main regulators of T_RM_ fate) showed less T-cell invasion and accumulation in contrast to wild-type mice, resulting in worse outcomes in the knockouts (41). T_RM_ cells can activate cross-presenting dermal DCs, resulting in priming of additional cytotoxic T cells against tumor-derived neo- and self-antigens (42). This T_RM_ cell-induced antigen spreading suppresses local and disseminated melanoma in mice (42). Taken together, this is strong experimental evidence that T_RM_ cells are essential for antitumor immunity in melanoma. CD103^+^CD69^+^ T_RM_ cells showed a high expression profile of immune checkpoint proteins and were located within the tumor tissue of melanoma patients, suggesting them to be an ideal subpopulation of T cells to be reinvigorated by ICI (35). In fact, T_RM_ cells were more efficient predictive biomarkers for the response to ICI than general CD8^+^ TILs in melanoma (43). In addition, within tumors, CD45RO^+^CD69^+^CCR7^−^ T_RM_ phenotypes were observed in 60% of CD8^+^ T cells and 50% of CD4^+^ T cells (35). Responders to ICI present an abundance of T_RM_ cells in pretreatment melanoma tissue (44). In particular, CD8^+^/CD4^+^EOMES^+^CD69^+^CD45RO^+^ subpopulations were expanded in responder patients. Also, a gene expression signature obtained from tumor biopsies containing the gene (ITGAE) encoding for CD103 among other TIL-related genes (CD8A, CD8B, ITGAE [CD103], PDCD1 [PD-1], CCL5, CXCL13, and IL2) was associated with better outcomes to anti-PD1 therapy (44). Interestingly, CD45RO^+^ and EOMES^+^ memory T cells measured in baseline melanoma tissue also highly expressed CD69 and CD103. They were more abundantly found in responder patients, further emphasizing the predominant role of T_RM_ cells as determinants of response and non-response (44). Another study in human melanoma samples found that 30% of the CD8^+^ T cells are T_RM_ cells positive for CD103 and CD69 (43). These T_RM_ cells showed moderate expression of granzyme B, CD137, and HLA-DR. CD103^+^CD69^+^ T_RM_ cells expressed checkpoint molecules and thus expanded under therapy (43). Local IL-15 highly correlated with T_RM_ cell numbers. IL-15 blocks tissue-egress signals on human T cells and induces the expression of CD69 (43). T_RM_ cells can also co-express CD49. CD49^+^ T_RM_ cells exhibited superior effector function and correlated with survival in a melanoma mouse model (45) (**Table 1**).

**Table 1:** Overview of current literature of tissue-resident memory T cells in melanoma.

Study	Species	ICI	T_RM_ cells	T_RM_ cell feature	Locations
(33)	Mice	Yes	T_CM_ cells shift to become T_RM_ cells after tumor inoculation	T_RM_ cells express PD-1 and expand upon ICI	Primary and metastatic melanoma
(35)	Human	Yes	50%–60% CD4^+^/CD8^+^ T_RM_ cells in tumors	Expressing checkpoint molecules	Melanoma metastases
(36)	Mice	No	Tumor-specific epidermal CD103^+^CD69^+^ T_RM_ cells	Correlated with spontaneous disease control	Primary melanoma
(37)	Mice	No	CD69^+^CD62L^lo^ tumor-specific LN T_RM_ cells	Protection against melanoma seeding in LNs	Melanoma, lymph node
(43)	Human	yes	30% of CD8^+^ TILs were CD69^+^CD103^+^ tumor-resident T_RM_ cells	Expressed checkpoints, correlated with melanoma-specific survival	Primary and metastatic melanoma
(46)	Mice	No	Melanoma antigen-specific CD103^+^CD69^+^ T_RM_ cells	Critical for protection against melanoma rechallenge	Primary melanoma/vitiligo
(47)	Human	Yes	CD69^+^ T_RM_ cells	IFNG/TNF-high signature, durable response	Metastatic melanoma/vitiligo
(42)	Mice/human	No	CD103^+^CD69^+^ T_RM_ cells	Antigen spreading via dermal DCs, recruiting more cytotoxic TILs	Melanoma, lymph node
(48)	Human	No	CD103^+^CD69^+^ T_RM_ cells	T_RM_ cells in metastatic melanoma > primary melanoma > nevi or healthy skin	Primary and metastatic melanoma, nevi, healthy skin
(45)	Mice and human	No	CD49^+^ T_RM_ cells	CD49a-expressing TIL co-express CD69 and CD103, improve survival	Primary and metastatic melanoma

## Immune-related adverse events and tissue-resident memory T cells

**Figure 1:**
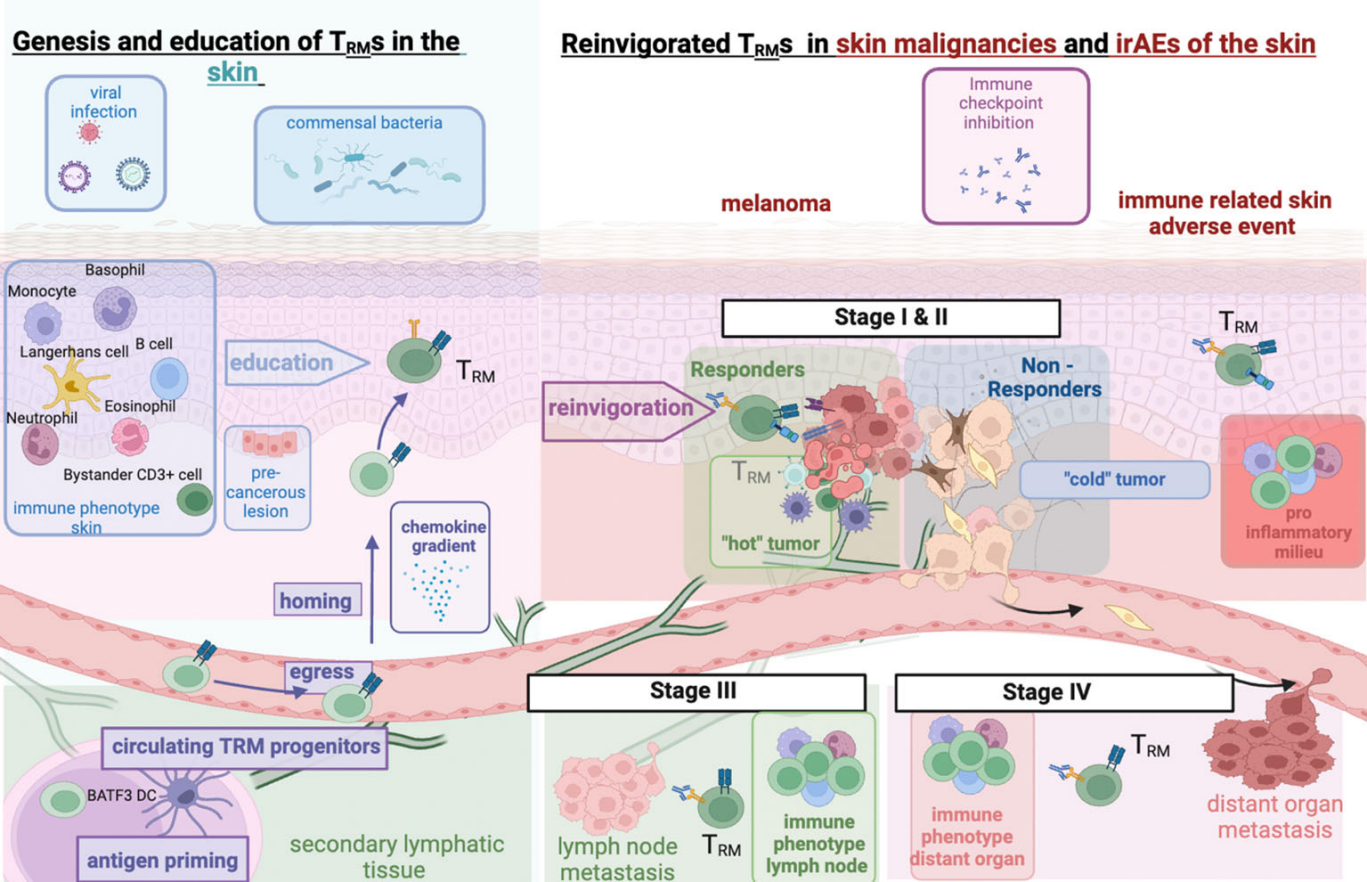
T_RM_ interaction in healthy skin and in ICI-treated melanoma and cutaneous irAEs. Left: Genesis of T_RM_ in healthy skin and impact of prior inflammation through commensal skin microbiota or prior immune responses to viral infections; right: immune checkpoint inhibition reinvigorates preformed T_RM_ cells. Malignancies undergo metastatic invasion with interaction with differentially shaped immune micromilieus in lymph nodes or distant organs. Expansion of activated T_RM_ cells to different cancer sites or off-target tissue sites (irAEs) upon ICI. Figure created with BioRender.

Although ICI can provoke a durable response for patients with advanced cancer, irAEs are challenging and limit the beneficial effects of treatment (49). They can be clinically categorized by the severity of the inflammatory side effects of ICI. IrAEs range from common and less severe cutaneous events like morbilliform or lichenoid rashes and pruritus to more severe toxicities such as dermatomyositis, Steven-Johnson syndrome, endocrinological dysfunction like diabetes, hepatitis, or myocarditis (50, 51). By clinical observation, the organ specificity of irAEs differs between CTLA-4 inhibitors and PD-1/PD-L1 inhibitors. For instance, patients treated with CTLA-4 inhibitors are more often affected by irAEs of higher severity like colitis and hypophysitis, whereas patients treated with PD-1/PD-L1 axis inhibitors more often develop pneumonitis and thyroiditis (52). Vitiligo is a side effect of ICI treatment specific to melanoma. Its association with a favorable outcome reveals the cross-reactivity of reinvigorated T cells (53, 54). In autoimmune vitiligo, CD8^+^ T_RM_ cells are recruited via CXCL9 and CXCL10 and result in melanocyte damage by secreting granzyme B, perforin, and IFN-γ (55). In mice, T_RM_ cells within vitiligo-affected tissue are also specific for melanoma antigens (46). These T_RM_ cells were not only critical for vitiligo lesions but also for maintaining antitumor immunity (46). In melanoma patients with response to immunotherapy and vitiligo, long-term antitumor immunity even up to 9 years after ICI was mediated via T_RM_ cells with high expression of IFN-γ and TNF-α (47). Interestingly, also other cutaneous irAEs and irAEs in general correlated with response to immunotherapy across cancers (56, 57). Recent studies of the cellular fraction in irAE-affected tissue revealed the abundance of T_RM_ cells in irAE colitis and irAE dermatitis (8–10). In various cutaneous irAEs, CD4^+^ and CD8^+^ T_RM_ cells were expanded and produced IFN-γ and TNF-α, arguing for Th1/Tc1 polarization (8). Downstream of IFN-γ, the CXCR3 ligands CXCL9–11 were upregulated, potentially recruiting more circulating T cells to the tissue. Furthermore, T_RM_ cells highly expressed inhibitory checkpoint molecules such as PD-1, CTLA-4, LAG-3, TIM-3, or TIGIT in irAE dermatitis. A similar expression pattern was seen in CD4^+^ and CD8^+^ T_RM_ cells of irAE colitis (8, 10). Upregulated checkpoints argue for potential reactivation and expansion of T_RM_ cells in off-target tissue during ICI (**Figure 1**). In addition, local IL-15 expression was also upregulated, corresponding with the high T_RM_ cell levels (8).

## Discussion

We are still missing reliable and mechanistically motivated tissue-based biomarkers that guide ICI at different stages of melanoma therapy. The tumor-intrinsic risk of recurrence in stage I–III melanomas can be classified with an immunohistochemical signature consisting of Bax, Bcl-X, PTEN, COX-2, β-catenin, MTAP, and CD20 (58–60). This signature, however, does not contain markers relevant to T cells which are the main target of ICI. CXCR3 ligands such as CXCL9 and CXCL10 as indirect markers of T-cell infiltration have been identified in the past as indicating a response to ICI in stage IV melanoma (61, 62). Lately, T_RM_ cells have become a subject of special interest and intensive research due to their unique combination of cytotoxic potential, combined with checkpoint protein expression, and their localization and high prevalence in tumor tissue (35). Microenvironmental factors, viruses, or microbiota can shape the appearance of T_RM_ cell pools (12). They have been found to patrol various organs such as the brain, intestine, skin, or even the heart (11, 63). T_RM_ cells are capable of plasticity and gene signature switches in agile and cytotoxic phenotypes (with cytolytic molecules) or endurance and senescence-like cell states in the absence of sustained antigen presentation (11). T_RM_ cells have proven to be strong predictors of survival across tumor entities (2–6). Specifically, in cancers treated with immunotherapy such as melanoma, they are a highly relevant target cell population because they express inhibitory checkpoint molecules (35). Melanoma often appears as a high metastatic entity and tends toward invasiveness and distant organ metastasis. The first results in mice have shown that T_RM_ cells can also colonize tumor-draining lymph nodes of melanoma patients and prevent metastatic spread from there (37). Investigation of the role of T_RM_ cells in tumor surveillance in disseminated tumor stages under immunotherapy is an urgent clinical question. Recent development and expansion of approval of pembrolizumab in the stage II setting of melanoma raises the question for assessing the T_RM_ profile and functional capacities in micrometastasis and MRD. Metastatic tumors face an altered immunologic niche. Adaptive immunity is confronted with transformed tumor cells undergoing epithelial–mesenchymal transition (EMT) (64). T_RM_ cells are thought to diminish proliferating epithelial-like tumor cells with high turnover and expression of epithelial markers (40). The prognostic role of T_RM_ cells within primary melanoma tissue and tumor-draining lymph nodes could become extremely interesting for the neoadjuvant ICI regimens and potentially guide neoadjuvant versus surgical approaches. In oral cancer patients, CD8^+^ TILs that clonally expanded during neoadjuvant ICI showed a cytotoxic T_RM_ cell phenotype, underlining the capacity for a rapid response of pre-existing T-cell clones (3). Those treatment-expanded T-cell clones in responding patients also recognized self-antigens such as the cancer-specific antigen MAGEA1 (3). Melanoma patients with vitiligo or other (cutaneous) irAEs tend to have a better outcome than others (56, 57). We hypothesize that Th1/Tc1-polarized T_RM_ cells that trigger cutaneous irAEs such as vitiligo can also be responsible for tumor cell killing through the production of effector molecules across melanoma stages I–IV. It is conceivable, downstream from IFN-γ-producing T_RM_ cells, that other circulating T cells are recruited to the tissue via CXCL9 and CXCL10 gradients (62). Exhausted T cells within melanoma tumors also produce CCL4 and CXCL13, which in turn recruit other relevant immune cells from the circulation such as dendritic or B cells, potentially resulting in tertiary lymphoid structures (74). Treating toxicity, systemic inhibition of Tc1/Th1 T_RM_ cells or corresponding cytokines such as IFN-γ might abrogate immunotherapy efficacy and should only be explored for severe irAEs. However, local and more targeted control of cutaneous irAEs might be achieved by topical treatment with JAK inhibitors instead of glucocorticoids without negatively affecting systemic antitumor immunity. In summary, T_RM_ cells can function as biomarkers for antitumor immunity and ICI toxicity and have to be targeted with caution. T_RM_ cells should be exploited as indicators for promising neoadjuvant ICI in melanoma and tested in larger trials.

## Author contributions

RR: Conceptualization, Visualization, Writing – original draft, Writing – review & editing, Methodology, Project administration. BD: Visualization, Writing – original draft. AE: Writing – review & editing. JH: Writing – review & editing.
